# Serum and Salivary Amylase Variations During Exercise Testing in Athletes and Their Correlation with Cardiovascular Parameters—A Pilot Study

**DOI:** 10.3390/medicina62071219

**Published:** 2026-06-23

**Authors:** Cezar Honceriu, Alexandru Dan Costache, Beatrice Aurelia Abălașei, Alin Ciobică, Alexandra Maștaleru, Andrei Drugescu, Diana Elena Cosău, Minerva Codruța Bădescu, Iulia Cristina Roca, Andreea Rotundu, Ovidiu Mitu, Irina Iuliana Costache Enache, Maria Magdalena Leon, Florin Mitu, Mihai Roca

**Affiliations:** 1 Faculty of Physical Education and Sports, “Alexandru-Ioan Cuza” University, 700115 Iasi, Romania; cezar.honceriu@uaic.ro (C.H.); beatriceabalasei@yahoo.com (B.A.A.); alin.ciobica@uaic.ro (A.C.); 2 “Grigore T. Popa” University of Medicine and Pharmacy, 700115 Iasi, Romania; alexandra.mastaleru@gmail.com (A.M.); andreidrugescu@yahoo.com (A.D.); e_cosau@yahoo.com (D.E.C.); minerva.badescu@umfiasi.ro (M.C.B.); iuliaroca@yahoo.com (I.C.R.); andreea.rotundu@gmail.com (A.R.); mituovidiu@yahoo.co.uk (O.M.); irina.costache@umfiasi.ro (I.I.C.E.); leon_mariamagdalena@yahoo.com (M.M.L.); mitu.florin@yahoo.com (F.M.); roca2m@yahoo.com (M.R.); 3 Clinical Rehabilitation Hospital, 700661 Iasi, Romania; 4 “St. Spiridon” Emergency County Hospital, 700111 Iasi, Romania; 5 Romanian Academy of Medical Sciences, 030167 Bucharest, Romania; 6 Romanian Academy of Scientists, 030167 Bucharest, Romania

**Keywords:** amylase, physical activity, athletes, cardiopulmonary exercise testing

## Abstract

*Background and Objectives:* During intense bouts of physical activity, the body of athletes is subjected to stress and sometimes this can lead to adverse events such as injuries or more severe organ dysfunction, like sudden cardiac death. Several markers are being studied to properly assess the level of physical stress that exercises have on the body and one of them is amylase. *Materials and Methods:* We evaluated 19 licensed football players using basic cardiovascular procedures, i.e., resting 12-lead electrocardiogram (ECG) and trans-thoracic echocardiography (TTE) and performing a cardiopulmonary exercise testing (CPET). Resting (T0) serum and salivary amylase levels were measured, as were immediately post-effort (T1) serum values and 10 min (T2) and 30 min post-CPET (T3) salivary values. Results: Both serum and salivary levels showed correlations with several TTE and CPET parameters. Only T2 salivary amylase levels did not show any correlations with the other parameters, while also no correlations could be established between serum and salivary determinations. *Conclusions:* Serum and salivary amylase determinations show potential in athlete evaluation even from a cardiovascular risk standpoint since they displayed several correlations with both TTE and CPET parameters, but as part of a more complex protocol. Salivary determinations cannot fully substitute serum measurements. Further studies on larger groups are required.

## 1. Introduction

Intense physical activity, despite its recognized benefits, is also perceived as a stress factor, especially when discussing competitive athletes. Therefore, when exposed to such situations, the body will begin secreting several hormones which can reflect its impact, commonly described as “stress hormones”. Among these is alpha-amylase, which is secreted because of the stimulation of the sympathetic adrenomedullary system and can be measured in subjects both from blood and saliva [1,2,3,4,5].

Compared to cortisol, salivary amylase levels increase faster and would be a more suitable and earlier marker of stress, as per several studies. This is possibly linked to a speedier response of the aforementioned sympathetic adrenomedullary system, which leads to the release of amylase, than that of the hypothalamic–pituitary–adrenocortical system, which is responsible for cortisol secretion [6,7,8].

Another important distinction between the two is that cortisol is linked more to emotional and unmanageable perceived situations (e.g., the stress of performing in front of audiences), while amylase is released in response to a controllable setting (e.g., the impact of physical activity itself on the body) [2].

Apart from monitoring training in athletes and their response during competition, we should also consider the possibility of major adverse cardiovascular events (MACE) occurring. Even though athletes are considered healthy and are often not documented with any pathologies, they are still at risk of such events, the most feared being sudden cardiac death. In 2020 the European Society of Cardiology issued its first edition of the Sports Cardiology Guidelines with several screening recommendations, even for apparently healthy individuals. At the moment it is advised to use the patient history, physical examination and resting electrocardiogram in apparently healthy young individuals, while more complex procedures such as stress testing and biomarkers are reserved for at-risk individuals [9].

However, low study numbers and low numbers of participants in the existing publications regarding athletes and screening, together with the need for further studies, have led us to integrate a full cardiovascular evaluation in our current investigation. This is especially since MACE do not only concern master athletes but also younger professional sportsmen, some of whom were victims of such events [9]. All things considered, our primary endpoint was to assess the variations in serum and salivary amylase in relation to physical effort in athletes.

Secondly, we conducted this study to evaluate the potential of certain usual markers, i.e., the dynamics of both serum and salivary amylase in athlete evaluation, especially their correlation with cardiovascular parameters and their potential to be further studied as markers for assessing risk of adverse events during physical activity, along with their possible integration in future screening protocols. We also wanted to compare serum and salivary determinations and whether the non-invasive salivary sampling could fully substitute the invasive blood sampling, given how many patients prefer non-invasive procedures for specimen collecting.

Our hypotheses included that amylase levels would increase after physical stress and that serum and salivary values would correlate, while these variations would also be in relation to cardiac parameters from transthoracic ultrasound and performance parameters from CPET.

Since this is a pilot study, we consider it could potentially encourage further studies to be conducted on larger groups to ascertain these findings.

## 2. Materials and Methods

### 2.1. Experimental Approach

We approached a comprehensive cardiometabolic assessment of a total of 19 professional football players, focusing on cardiopulmonary exercise testing (CPET) and measuring serum and salivary levels of alpha-amylase to evaluate the body’s stress response during intense physical activity as the highlight investigative procedures. The study adhered to the clinical research guidelines of the Helsinki Declaration of the World Medical Association, while the protocol received approval from both the Ethics Committee of the Clinical Rehabilitation Hospital in Iași, Romania (approved on 24 March 2021) and the Ethics Research Committee of the “Grigore T. Popa” University of Medicine and Pharmacy in Iași, Romania (approval number 72, dated 25 April 2021).

### 2.2. Participants and Protocol

We evaluated a total of 19 male professional football players licensed in various clubs in the region, aged between 18 and 20 years (average age 18.47 ± 0.841). All of them were playing at the same level, with similar years of experience and had undergone similar training regimens (type of training, load, duration) for years, in order to ensure the uniformity of the cohort. We did not consider playing positions as a criterion for inclusion into the study.

Those who were injured at the time, in post-injury recovery, or who declined participation were excluded from the study. Along with those, we excluded players who were taking medication at the time or who were known to have subclinical conditions.

To ensure accurate results and a similar starting point, as a pre-protocol routine, we asked them to avoid intense training or matches for at least 24 h before the tests. Additionally, they were instructed not to eat on the morning of the saliva sample collection and to refrain from brushing their teeth or using mouthwash. Also, to ensure uniformity, we performed all testing in the interval of 8 a.m.–11 a.m. when possible for all participants.

They were admitted to the Cardiology Clinic of the Rehabilitation Hospital for day hospitalization in August and September 2021. All informed consent forms regarding the procedures and their involvement in the study were signed before any examinations began.

### 2.3. Initial Evaluation

After collecting the resting serum and saliva cortisol samples, all participants had their resting blood pressure and heart rate values measured, followed by a resting 12-lead electrocardiogram (ECG) and a standard transthoracic echocardiographic (TTE) evaluation.

The parameters collected for every subject from TTE were as follows:

- EDV—end-diastolic volume;
- ESV—end-systolic volume;
- SV—stroke volume;
- EF—ejection fraction;
- FS—fractional shortening;
- SI—stroke-volume index;
- IVSTd—interventricular septum thickness at end-diastole;
- LVIDd—left ventricular internal dimension at end-diastole;
- LVPWTd—left ventricular posterior wall thickness at end-diastole;
- IVSTs—interventricular septum thickness at end-systole;
- LVIDs—left ventricular internal dimension at end-systole;
- LVPWTs—left ventricular posterior wall thickness at end-systole;
- LV MASSd—left ventricular mass at end-diastole;
- LV MASSd Index—left ventricular mass at end-diastole adjusted to body surface index;
- LV MASSs—left ventricular mass at end-systole;
- LV MASSs Index—left ventricular mass at end-systole adjusted to body surface index;
- E Vel—peak velocity of early diastolic mitral annular motion as determined by pulsed wave Doppler;
- A Vel—peak velocity of diastolic mitral annular motion as determined by pulsed wave Doppler;
- E/A—ratio of E to A;
- A/E—ratio of A to E;
- DcT—deceleration time MV area;
- PHT—mitral valve area at pressure half time;
- PHT—pressure half time;
- LVOT Diam—left ventricular outflow tract diameter;
- Ao Diam—aortic annulus diameter;
- LA Diam—left atrium diameter;
- LA/Ao—ratio of the left atrial dimension to the aortic annulus dimension.

Also, medical history was taken, and the basic physical examination was performed.

### 2.4. Cardiopulmonary Exercise Testing

CPET was conducted using the BTL CardioPoint software (version 2.32) and compatible equipment to evaluate the athletes’ functional capacity. The protocol involved a progressive maximal symptom-limited test on a cycle ergometer, starting at 15 Watts with an increase of 12.5 Watts every 30 s, lasting about 10 to 12 min, followed by a 10 min resting period.

CPET enabled us to record certain parameters such as the following:

- Maximum work rate (WR) in Watts and as a percentage of the predicted value (WR%);
- Peak oxygen uptake (peak VO_2_) in milliliters per minute and as a percentage of the predicted value (%VO_2_ max);
- Carbon dioxide output (VCO_2_) in milliliters per minute;
- Oxygen uptake at the anaerobic threshold (AT) in milliliters per minute and normalized by body weight (milliliters per minute per kilogram) to fit the subjects into the Weber class, using the Wasserman curve on ventilatory equivalents (VE/VO_2_);
- The oxygen pulse (O_2_ pulse—mL/beat) indicated the maximum stroke volume during effort;
- Metabolic efficiency as the ratio of the increase in VO_2_ to the rate of work rate increase (VO_2_/WR), reflecting muscle oxygen extraction during exercise.

Blood pressure was measured every two minutes using the auscultatory method, with a 12-lead ECG recorded in real-time.

The severity of perceived exhaustion was assessed using a 6–20 Borg scale to determine exercise intensity.

The test would be stopped in the following situations:

1. At the participant’s request;
2. Upon symptoms or fatigue occurrence;
3. If systolic blood pressure exceeded 220 mmHg;
4. Diastolic blood pressure exceeded 120 mmHg;
5. Suggestive ischemic ECG patterns during stress.

### 2.5. Amylase Determinations

For measuring the resting laboratory parameters, peripheral venous blood samples were collected before performing the CPET investigation (T0). Also, specimens were gathered for determining salivary alpha-amylase levels at rest (T0) using specialized saliva collection kits. After completing the CPET, another blood sample was taken to measure immediate post-exercise alpha-amylase levels (T1). Blood was drawn from antecubital veins using dedicated vacutainers.

Further saliva samples were collected at 10 min (T2) and 30 min (T3) after the CPET using the same specialized saliva collection kits to determine salivary alpha-amylase levels at a total of three different timepoints (T0, T2, T3). The measurements were carried out using the ELISA procedure, with a semi-automated ELISA model UT6500 by MRC and DiaMetra (06038 Spello–Perugia Italy) kits.

The number of determinations, as well as their timepoints for both serum and salivary samples, were chosen based on the dynamics of amylase as published, as well as other studies’ protocols. Several publications have said that in saliva, amylase levels start dropping after the 10 min interval, so we were still in the period of peak levels. Also, when taking saliva samples, the footballers had to hold a swab in their mouth for several minutes which was easier 10 min after the CPET, since the recovery period also incorporates two minutes of light-pedaling and eight minutes of resting blood pressure and ECG measurements [10,11].

Alpha-amylase determinations were performed on serum samples using the kit code AY8004, manufactured by Randox Laboratories Ltd., London, UK. For saliva samples, the Salivary A-Amylase Kinetic Enzyme Assay Kit, manufactured by Salimetrics, State College, PA 16803, USA, was used.

Serum samples were collected on vacutainers without additives, the serum was separated by centrifugation and stored at −80 degrees Celsius for 6 months. No preservatives were used. Saliva samples were stored at −80 degrees Celsius for 6 months.

### 2.6. Data Analysis and Statistics

Data analysis was conducted using SPSS version 20.0 (Statistical Package for the Social Sciences, Chicago, IL, USA). Continuous variables were reported as either means with standard deviations (SD) in the case of normal distribution or medians with interquartile ranges in the case of non-normally distributed data. Corresponding to the two scenarios, paired-groups analysis was done using the paired-samples *t*-test and the Wilcoxon signed-rank test, respectively. The Hodges–Lehmann estimator was used to calculate the point estimate of the median difference between the paired groups. To provide a 95% confidence interval for this median difference, bootstrapping was utilized due to its robustness against non-normality. Spearman correlation coefficients were calculated to assess relationships between continuous variables. A two-sided *p*-value of ≤0.05 was considered statistically significant. In order to decrease the risk of Type I errors, in case of multiple comparisons, *p*-value was adjusted by the Bonferroni correction method.

For the entire study group, statistical power analysis was performed using G*Power v.3.1.9.7.

## 3. Results

During the taking of the medical history and the physical examination, no significant findings were recorded.

The resting ECG recordings showed patterns such as right bundle branch block or QRS-complex hyper-voltage. However, these are considered normal encounters in competitive athletes; therefore, they did not hinder further examination.

TTE examination showed patterns of the athletes’ heart with uniformly increased wall thickness and enlarged cavities, without any significant pathological aspects. The values of all TTE parameters can be found in Table 1, shown as median and interquartile interval (see Table 1).

During CPET, no symptoms suggestive of myocardial ischemia were declared, nor were any ischemic or abnormal ECG patterns observed. The functional capacity of every subject was normal, as every one of them was placed into the A Weber class. Interestingly, there were several instances of an increase in systolic blood pressure to 220 mmHg which, according to the protocol used in the clinic, warranted the halting of the testing.

The values of all CEPT parameters can be found in Table 2, shown as median and interquartile intervals (see Table 2).

The values of all amylase determinations at any given timepoint can be found in Table 3, shown as median and interquartile intervals (see Table 3).

Considering the normal distribution of serum amylase, a paired-samples *t*-test was applied to compare paired samples at the two different timepoints. This proved a statistically significant increase in serum amylase (*p* = 0.025) from T0 to T1.

A Wilcoxon signed-rank test with continuity correction was conducted to compare paired salivary amylase samples at different timepoints, considering the non-normal distribution of this parameter.

There was no statistically significant difference in the median levels of T0 salivary amylase and T2 salivary amylase (V = 43, *p* = 0.067, 95% CI [−198.8, 2.5]). While the (pseudo) median difference was −154.7, the bootstrap 95% confidence interval for the median difference was (−344.9, 0.0), suggesting the difference is not significant.

The difference between T2 salivary amylase and T3 salivary amylase was not statistically significant (V = 102, *p* = 0.0832, (pseudo)median difference = 136.55). A 95% bootstrap confidence interval for the median difference (R = 10,000) was found to be [0.00, 69.89], indicating a marginal difference between the groups.

There was also no statistically significant difference in the median saliva levels at T0 and T3 timepoints (V = 83, *p* = 0.9306, pseudo-median difference = −0.56). A nonparametric bootstrap (10,000 samples) produced a 95% percentile confidence interval for the median difference of (−14.73, 18.39), supporting the conclusion that there is no significant change.

Although not statistically significant, salivary amylase showed an increase from T0 to T2 and a decrease from T2 to T3 (see Table 4).

Serum amylase collected at rest (T0) showed correlations with the following parameters:

- Aortic diameter (Ao Diam) from TTE (*p* = 0.019);
- Peak oxygen consumption (peak VO_2_) during CPET (*p* = 0.020);
- Percentage achieved from the predicted maximum oxygen consumption (%VO_2_ max) during CPET (*p* = 0.033);
- Metabolic efficiency (VO_2_/WR) during CPET (*p* = 0.006);
- Oxygen pulse (O_2_ pulse) (*p* = 0.037);
- Maximum systolic blood pressure (SBP max) during CPET (*p* = 0.025);
- Post-effort (T1) serum amylase (*p* = 0.000) (see Table 5).

After Bonferroni correction, it can be observed that only VO_2_/WR (*p* = 0.042, r = 0.605—large effect size) and T1 serum amylase (*p* = 0.000, r = 0.923—large effect size) maintained their correlation to T0 serum amylase (see Table 5).

Serum amylase collected post-effort (T1) showed correlations with the following parameters:

- Ao Diam from TTE (*p* = 0.017);
- VO_2_/WR during CPET (*p* = 0.009);
- SBP max during CPET (*p* = 0.022);
- T0 serum amylase (*p* = 0.000) (see Table 6).

After Bonferroni correction, it can be observed that only VO_2_/WR (*p* = 0.036, r = 0.582—large effect size) and T0 serum amylase (*p* = 0.000, r = 0.923—large effect size) maintained their correlation to T1 serum amylase (see Table 6).

Salivary amylase collected at rest (T0) showed correlations with the following parameters:

- Weight of the subject (*p* = 0.004);
- E wave velocity (E vel) from TTE (*p* = 0.016);
- E/A ratio from TTE (*p* = 0.034);
- 30 min post-effort (T3) salivary amylase (*p* = 0.044) (see Table 7).

After Bonferroni correction, it can be observed that only the weight of the subjects maintained its correlation to T0 salivary amylase (*p* = 0.016, r = 0.625—large effect size) (see Table 7).

Salivary amylase collected 30 min post-effort (T3) showed correlations with the following parameters:

- Weight of the subject (*p* = 0.017);
- E/A ratio from TTE (*p* = 0.018);
- Oxygen volume at the anaerobic threshold point (VO_2_-AT) during CPET (*p* = 0.049);
- VO_2_-AT/body weight during CPET (*p* = 0.006);
- Resting (T0) salivary amylase (*p* = 0.044) (see Table 8).

After Bonferroni correction, it can be observed that only VO_2_-AT/body weight maintained its correlation to T3 salivary amylase (*p* = 0.03, r = 0.609—large effect size) (see Table 8).

No correlations could be established between serum and salivary amylase determinations, since any given timepoint values showed no *p*-values below 0.05 when correlated to the others.

**Figure 1:**
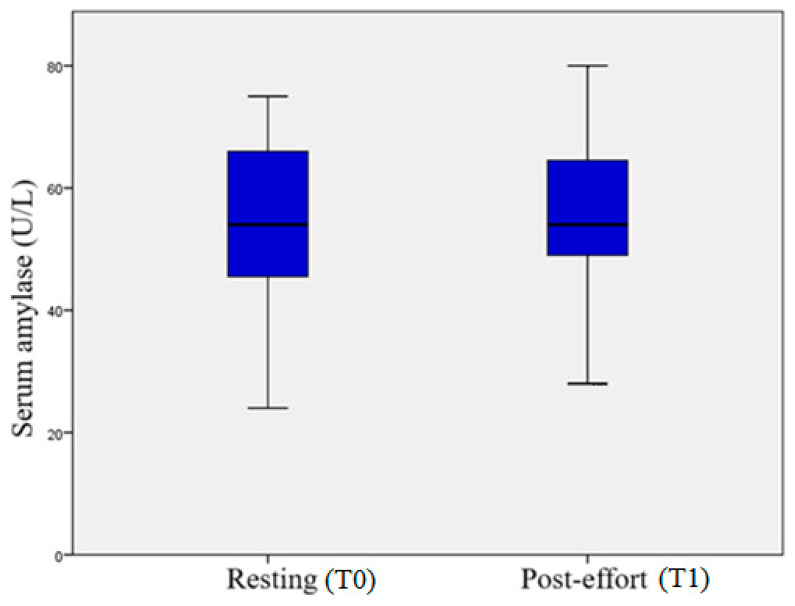
Post-effort variation in serum amylase.

Serum amylase presented a statistically significant increase (*p* = 0.025) from T0 to T1 (see Figure 1 and Table 9).

**Figure 2:**
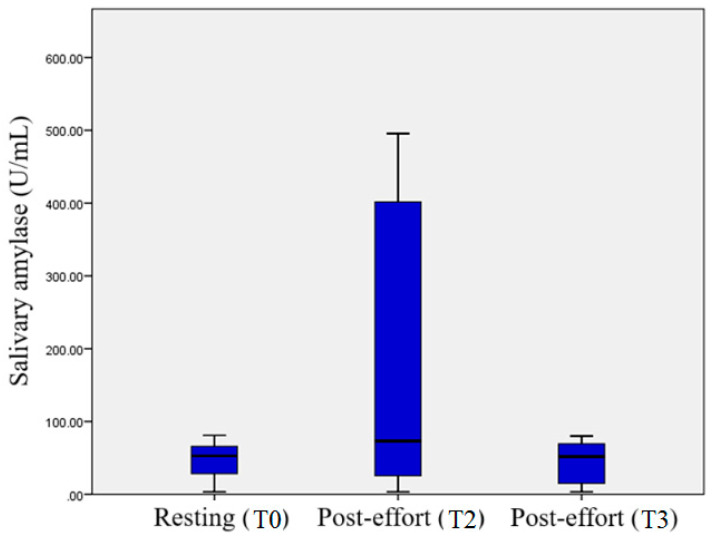
Post-effort variation in salivary amylase.

Salivary amylase showed an increase from T0 to T2 and a decrease from T2 to T3, although in both cases this was not statistically significant (*p* = 0.064 and *p* = 0.079, respectively). No significant difference was found between T0 and T3 (see Figure 2 and Table 9).

## 4. Discussion

Amylase has also been previously studied on large groups of healthy individuals which helped characterize its dynamics especially related to physical effort. We chose to focus on studies involving similar groups (athletes), similar sample sizes or comparable circumstances (dynamics of amylase related to physical stress) [12,13,14,15,16,17].

Several previous studies have reported increased levels of amylase prior and during competitive season. The fact that it showed increased values pre-competition is linked to the possible emotional component. This is also supported by the significantly higher levels which were observed during competition rather than during training and by the significant drop to baseline levels in only 30 min after physical effort [1,18,19,20,21]. Indeed, this can also be an aspect to consider when analyzing our results, since in this case we evaluated athletes outside the competitive season and after a short rest from training.

The activity of salivary alpha-amylase can also be affected by dietary choices. Research conducted by Lu et al. revealed that consuming 400 mL of alkaline water alongside 0.15 g/kg of L-glutamine before training led to an increase in salivary α-amylase activity, which aided the recovery process [22].

Salivary alpha-amylase was evaluated in a study of breast cancer survivors. Normally, after waking up, levels should decrease in 30 min. In these patients, higher diurnal levels were observed especially after chemotherapy sessions. Training regimens using high-intensity interval training showed an improvement in alpha-amylase levels upon waking up [23].

In a previous publication, we evaluated serum and salivary cortisol variations in the same cohort of subjects. When analyzing the amylase values, we could establish several correlations between T3 salivary amylase and T2 salivary cortisol (*p* = 0.026) [24].

The utility of amylase and cortisol in athletes has also been assessed in many fields of medicine, such as neurology. In 2020, Mehrsafar et al. published their work on transcranial direct current stimulation of the dorsolateral prefrontal cortex as a potential modulation of stress and anxiety caused by competition. Salivary alpha-amylase and cortisol were used as markers of stress and their levels indeed showed significant decrease, suggesting a potential role in improving competitive performance [25].

This aspect of anxiety in performance sports was also studied by Lim in archery. Anxiety levels were quantified using salivary amylase and cortisol and lower levels of both markers were associated with increased performance [26]. However, this is a different type of physical activity from the one which our study group practiced.

An important aspect to consider when analyzing serum and salivary variation is the influence that other factors may have exhibited, such as nutrition and hydration, apart from the physical or mental stress. As Backes et al. showed, conditioned hydration influenced cortisol levels but not amylase [27]. Because of this and to not interfere with salivary sampling, we recommended minimum hydration prior to CPET and to avoid brushing or using mouthwash in the morning.

Several sportsmen choose to consume ergogenic aids such as caffeine or β-alanine to properly manage stressful periods, and these also induce changes in both markers, yet few studies have measured both and correlated them [28]. We also recommended to our study group to avoid such substances.

Also, apart from diet and training regimens, a significant and often understated impact is that of sleep patterns especially in young adults. This has been highlighted by Haddad et al. as they showed how in this population, sleep, physical activity and academic performance are interconnected and may influence each other [29].

As mentioned prior, we chose subjects of similar experience, playing level and chronic training exposure. This is important for the uniformity of the study group, as the intensity of the training sessions may impact the physical performance of certain acts (e.g., cycling, running, breathing) and, consequently, the variation in the studied parameters. This has been explained by Padulo et al. in their study on running gait variability and how it is impacted by training. It is an indicator of adaptation to training load, as it is correlated to fatigue in long-distance runners [30].

Considering the timepoints of amylase sampling there is this discrepancy between serum samples which were collected immediately post-effort (T1) and saliva samples which were taken after 10 (T2) and 30 min (T3) respectively. These were selected after several publications have shown the dynamics of amylase in both serum and saliva related to physical stress. In saliva, amylase levels start dropping after the 10 min interval, so we were still in the period of peak levels. Also, when taking saliva samples, the footballers had to hold a swab in the mouth for several minutes which was easier 10 min after the CPET, since the recovery period also incorporates two minutes of light-pedaling and eight minutes of resting blood pressure and ECG measurements [10,11].

As a practical aspect of the study, T0 salivary and serum amylase values together with T1 serum and T3 salivary are useful in assessing the impact of physical activity in athletes since they showed several correlations with CPET. Since T2 salivary amylase showed no correlations, it did not prove to be as useful as the other determinations. This however should be considered in the context of the limitations of our study which we have discussed, and it should be a focal point of future studies on larger groups—whether to investigate the utility of sampling salivary amylase at this timepoint.

In the context of the above-mentioned correlations, serum and salivary amylase provide valuable indicators of subject performance and functional capacity, particularly through correlations with CPET parameters (i.e., VO_2_/WR, VO_2_-AT/body weight). However, interpreting these associations requires caution, as correlation does not equate to causation.

From a clinical point of view, these results show that amylase may reflect acute autonomic and cardiovascular adaptations to exercise, rather than being a stand-alone diagnostic marker. Correlations with CPET parameters such as metabolic efficiency, systolic blood pressure or the oxygen uptake at the anaerobic threshold divided by body weight are explained by the connection between amylase levels and the sympathetic adrenomedullary activation during stress. This leads to higher metabolic and circulatory drive.

As previously shown, the Bonferroni tests enabled to correct *p*-values and fewer correlations remained after they were applied. This highlights the need for a larger study group to confirm the initial correlations, while also underscoring their potential in future studies.

At the moment, corrected *p*-values using Bonferroni tests are highlighting potential aims of future research, while the previously uncorrected values have enabled us to generate further hypotheses.

In a practical clinical setting, salivary determinations are easier for the medical personnel (from the technical standpoint) as well as for the patients (especially if they declare a fear of needles). However, no correlations could be established between serum and salivary amylase values, meaning that salivary determinations cannot substitute blood sampling.

For clinicians involved in sports medicine or preparticipation cardiovascular assessment, these measures may therefore be considered as complementary biomarkers within a broader protocol that already includes history, examination, ECG, echocardiography, and functional testing, especially when trying to characterize physiological adaptation to exertion.

However, the absence of significant correlations between serum and salivary measurements indicates that salivary sampling cannot yet replace blood testing, and the small pilot sample, multiple-comparison sensitivity, and restricted population all mean that the present data are not sufficient to support confident routine clinical or policy-level implementation without additional confirmatory research.

There are two main limitations of this study which are worth mentioning. Firstly, there was a small number of participants which warranted the use of non-parametric statistical analysis. It is therefore recommended to continue this study on a larger population to confirm the presented results, as shown by other studies on smaller groups [31].

The sample size of 19 subjects restricted us to non-parametric statistical tests. However, many studies conducted on athletes inherently have smaller cohorts compared to studies on clinical populations. Rather than confirming hypotheses, the primary value of this preliminary publication lies in its hypothesis generation, assessment of feasibility, and estimation of data variability. The effect sizes and standard deviations observed here will be critical for accurately designing and estimating the required sample sizes for future, larger-scale studies.

Secondly, a reevaluation of the athletes or more measurements at different timepoints would have given a better understanding of the dynamics of both serum and salivary amylase.

Also, given the above-mentioned publications, one major limitation of our study is the variation in amylase related to nutrition and anxiety. For this, we gave all participants the same pre-examination recommendations regarding diet and hydration.

Further limitations which may influence the results are the different timepoints for sampling serum and salivary amylase respectively, which makes a clear comparison difficult, along with testing the subjects outside the competitive season, adding to the risk of bias.

Considering the limitations of the study, it is important to address the discussion of only one parameter in this publication—amylase. The use of multiple markers has been suggested by Cardoso et al. as a means of better evaluating athletes and offering a better picture of organism adaptations. They also showed how caffeine is ergogenic for postural muscles, as it improved muscle activity and decreased the rate of perceived effort [32].

The study also raises the need for larger and adequately powered prospective studies that are needed to confirm which amylase sampling timepoints and methods are truly practical and useful, while the observed associations persist after considering factors previously mentioned such as hydration status, diet, sleep, anxiety, training load, and circadian variability.

Future studies should employ protocols focused on repeated measurements across training cycles, competitive versus non-competitive periods, and recovery windows shorter and longer than those used here to better describe the dynamics of both serum and salivary amylase. It could also be useful to compare different cohorts, including female athletes, athletes from distinct types of sports, athletes of different ages, and individuals with different cardiovascular risk profiles, to prove external validity and clinically meaningful reference ranges.

Mechanistic studies integrating amylase with other biomarkers of autonomic, endocrine, inflammatory, and myocardial stress may clarify whether amylase adds incremental value over established measures, while predictive studies should assess whether amylase-based models can improve risk stratification, screening efficiency, or monitoring of training adaptation. In parallel, methodological studies should determine whether simplified salivary protocols can be standardized sufficiently for field use, or whether serum assays remain necessary when more precise cardiovascular interpretation is required.

## 5. Conclusions

Serum and salivary amylase determinations show significant correlations with specific cardiopulmonary exercise testing parameters, holding great promise for screening athletes—especially in facilities where standard CPET equipment is unavailable. These markers are useful for estimating functional performance and reflecting the degree of cardiovascular adaptation athletes achieve through prolonged physical training.

Serum and salivary values did not correlate with each other; therefore, salivary measurements cannot fully substitute for blood sampling.

These are more useful in athlete evaluation as part of a larger and more complex protocol, involving other investigations.

However, the small number of subjects and determinations warrants further studies on larger groups. Since this is a pilot study, these preliminary results could potentially encourage further studies to be conducted on larger groups to ascertain these findings.

## Figures and Tables

**Table 1:** TTE parameters as median and interquartile intervals.

Parameter	Median	Interquartile Interval
EDV (Teich-mL)	114.400	101.900–127.200
ESV (Teich-mL)	33.00	29.80–36.40
SV (Teich-mL)	76.400	65.500–94.200
EF (Teich-%)	72.100	64.700–74.100
FS (%)	41.600	35.200–43.400
SI (Teich-mL/m^2^)	40.400	36.100–49.700
IVSTd (mm)	10.600	9.400–11.100
LVIDd (mm)	49.300	46.900–51.600
LVPWTd (mm)	10.00	9.40–11.10
IVSTs (mm)	13.500	12.300–15.800
LVIDs (mm)	29.30	28.10–31.10
LVPWTs (mm)	16.400	14.700–18.200
LV MASSd (ASE-g)	237.00	198.00–267.00
LV MASSd Index (ASE-g/m^2^)	123.8100	110.0000–142.0200
LV MASSs (ASE-g)	188.00	169.00–236.00
LV MASSs Index (ASE-g/m^2^)	99.4300	85.1900–131.0900
E Vel (cm/s)	79.300	67.900–85.200
A Vel (cm/s)	44.00	37.70–52.20
E/A	1.7800	1.5700–2.0600
A/E	0.5600	0.4900–0.6400
DcT (s)	0.24200	0.20400–0.31700
MVArea PHT (cm^2^)	3.10	2.37–3.86
PHT (s)	0.07100	0.06000–0.09300
LVOT Diam (mm)	25.700	24.400–26.700
Ao Diam (mm)	30.600	28.900–31.900
LA Diam (mm)	27.700	24.400–30.800
LA/Ao	0.9700	0.8100–1.0200

Abbreviations: EDV—end-diastolic volume; ESV—end-systolic volume; SV—stroke volume; EF—ejection fraction; FS—fractional shortening; SI—stroke-volume index; IVSTd—interventricular septum thickness at end-diastole; LVIDd—left ventricular internal dimension at end-diastole; LVPWTd—left ventricular posterior wall thickness at end-diastole; IVSTs—interventricular septum thickness at end-systole; LVIDs—left ventricular internal dimension at end-systole; LVPWTs—left ventricular posterior wall thickness at end-systole; LV MASSd—left ventricular mass at end-diastole; LV MASSd Index—left ventricular mass at end-diastole adjusted to body surface index; LV MASSs—left ventricular mass at end-systole; LV MASSs Index—left ventricular mass at end-systole adjusted to body surface index; E Vel—peak velocity of early diastolic mitral annular motion as determined by pulsed wave Doppler; A Vel—peak velocity of diastolic mitral annular motion as determined by pulsed wave Doppler; E/A—ratio of E to A; A/E—ratio of A to E; DcT—deceleration time MV area; MVArea PHT—mitral valve area at pressure half time; PHT—pressure half time; LVOT Diam—left ventricular outflow tract diameter; Ao Diam—aortic annulus diameter; LA Diam—left atrium diameter; LA/Ao—ratio of the left atrial dimension to the aortic annulus dimension.

**Table 2:** CPET parameters as median and interquartile intervals.

Parameter	Median	Interquartile Interval
Peak VO_2_ (mL/min)	3753.00	3202.00–3941.00
Peak VO_2_ body weight (mL/min/kg)	50.0634	45.5312–54.1926
%VO_2_ max	109.00	96.50–119.50
VO_2_@AT (mL/min)	2431.00	2103.50–2893.50
VO_2_@AT body weight (mL/min/kg)	34.1166	28.7952–42.6298
RER	1.1100	1.0600–1.1600
VE/VCO_2_	22.3500	21.2050–26.1200
ΔVO_2_/ΔWR (mL/min/Watt)	13.2000	12.0600–14.0800
WR max (Watt)	248.00	229.00–260.00
%WR max	86.00	82.00–88.50
O_2__pulse (mL/beat)	21.700	19.000–22.900
%O_2__pulse	134.3252	117.1846–141.7677
HR max (bpm)	167.00	163.00–177.50
%HR max	83.00	80.50–88.00
HR_rez (bpm)	107.00	99.00–112.00
SBP max (mmHg)	220.00	205.00–227.50
DBP max (mmHg)	85.00	80.00–85.00

Abbreviations: peak VO_2_—peak oxygen uptake; peak VO_2_ body weight—ratio of peak oxygen uptake to body weight; %VO_2_ max—percentage of maximum oxygen uptake from the predicted value; VO_2_@AT—oxygen uptake at the anaerobic threshold; VO_2_@AT body weight—ratio of oxygen uptake at the anaerobic threshold to body weight; RER—respiratory exchange ratio; VE/VCO_2_—ventilatory equivalent for carbon dioxide; ΔVO_2_/ΔWR—slope of the relation VO_2_–power in W; WR max—maximum load; %WR max—percentage of maximum load from the predicted value; O_2__pulse—oxygen pulse; HR max—maximum heart rate; %HR max—percentage of maximum heart rate from the predicted value; HR_rez—heart rate reserve; SBP max—maximum systolic blood pressure; DBP max—maximum diastolic blood pressure.

**Table 3:** Amylase determinations at different timepoints as median and interquartile intervals.

Parameter	Median	Interquartile Interval
T0 serum amylase (U/L)	54.00	45.00–68.00
T1 serum amylase (U/L)	54.00	49.00–66.00
T0 salivary amylase (U/mL)	52.74	25.7800–66.8400
T2 salivary amylase (U/mL)	73.170	21.350–405.000
T3 salivary amylase (U/mL)	51.8800	3.2800–70.3500

**Table 4:** Wilcoxon test for salivary amylase measurements.

	T2 Salivary Amylase-T0 Salivary Amylase	T3 Salivary Amylase-T2 Salivary Amylase	T3 Salivary Amylase-T0 Salivary Amylase
*p*-value	0.067	0.083	0.93

**Table 5:** T0 serum amylase correlations.

T0 Serum Amylase	r	p	*p* *
Ao Diam (mm)	0.531	0.019	0.133
Peak VO_2_ (mL/min)	0.530	0.020	0.140
%VO_2_ max	0.489	0.033	0.231
VO_2_/WR (mL/min/W)	0.605	0.006	0.042
O_2_ pulse (mL/b)	0.481	0.037	0.259
SBP max (mmHg)	0.513	0.025	0.175
T1 serum amylase	0.923	0.000	0.000

* *p*-value adjusted for multiple comparisons by the Bonferroni correction method.

**Table 6:** T1 serum amylase correlations.

T1 Serum Amylase	r	p	*p* *
Ao Diam (mm)	0.541	0.017	0.068
VO_2_/WR (mL/min/W)	0.582	0.009	0.036
SBP max (mmHg)	0.522	0.022	0.088
T0 serum amylase	0.923	0.000	0.000

* *p*-value adjusted for multiple comparisons by the Bonferroni correction method.

**Table 7:** T0 salivary amylase correlations.

T0 Salivary Amylase	r	p	*p* *
Weight (kg)	0.625	0.004	0.016
E vel	0.543	0.016	0.064
E/A	0.488	0.034	0.136
T3 salivary amylase	0.467	0.044	0.176

* *p*-value adjusted for multiple comparisons by the Bonferroni correction method.

**Table 8:** T3 salivary amylase correlations.

T3 Salivary Amylase	r	p	*p* *
Weight (kg)	0.359	0.017	0.085
E/A	0.537	0.018	0.09
VO_2_-AT (mL/min)	0.457	0.049	0.245
VO_2_-AT/body weight	0.609	0.006	0.03
T0 salivary amylase	0.467	0.044	0.22

* *p*-value adjusted for multiple comparisons by the Bonferroni correction method.

**Table 9:** Comparisons of serum and salivary amylase values at different timepoints (Data are presented as mean ± standard deviation, or median (interquartile range)).

**T0 serum amylase (U/L)**	**T1 serum amylase (U/L)**	*p*-value *
53.05 ± 14.34	55.42 ± 15.21	0.025
**T0 salivary amylase (U/mL)**	**T2 salivary amylase (U/mL)**	*p*-value **
52.74 (25.78–66.84)	73.17 (21.35–405.00)	0.067
**T2 salivary amylase (U/mL)**	**T3 salivary amylase (U/mL)**	*p*-value **
73.17 (21.35–405.00)	51.88 (3.28–70.35)	0.083
**T0 salivary amylase (U/mL)**	**T3 salivary amylase (U/mL)**	*p*-value **
52.74 (25.78–66.84)	51.88 (3.28–70.35)	0.930

* Paired samples *t*-test; ** Wilcoxon signed-rank test.

## Data Availability

Data is contained within the article.

## Abbreviations

The following abbreviations are used in this manuscript:

%VO_2_ max	Percentage of the predicted maximum oxygen uptake
%WR	Percentage of the predicted maximum work rate
Ao Diam	Aortic diameter
AT	Anaerobic threshold
CPET	Cardiopulmonary exercise testing
CWD	Continuous wave Doppler
E vel	E wave velocity
O_2_ pulse	Oxygen pulse
PWD	Pulse wave Doppler
RER	Respiratory exchange ratio
SBP	Systolic blood pressure
T0	Resting
T1	Immediate post-effort
T2	10 min post-effort
T3	30 min post-effort
TTE	Trans-thoracic echocardiography
VCO_2_	Carbon dioxide output
Peak VO_2_	Peak oxygen uptake
VO_2_/WR	Metabolic efficiency
WR	Maximum work rate
